# Perceptions of Health Co-Benefits in Relation to Greenhouse Gas Emission Reductions: A Survey among Urban Residents in Three Chinese Cities

**DOI:** 10.3390/ijerph14030298

**Published:** 2017-03-13

**Authors:** Jinghong Gao, Guozhang Xu, Wenjun Ma, Yong Zhang, Alistair Woodward, Sotiris Vardoulakis, Sari Kovats, Paul Wilkinson, Tianfeng He, Hualiang Lin, Tao Liu, Shaohua Gu, Jun Wang, Jing Li, Jun Yang, Xiaobo Liu, Jing Li, Haixia Wu, Qiyong Liu

**Affiliations:** 1 State Key Laboratory of Infectious Disease Prevention and Control, Collaborative Innovation Center for Diagnosis and Treatment of Infectious Diseases, National Institute for Communicable Disease Control and Prevention, Chinese Center for Disease Control and Prevention, Beijing 102206, China; gaojinghong2007@126.com (J.G.); wangjun@icdc.cn (J.W.); lijingsddx@126.com (J.L.); smart_yjun@163.com (J.Y.); liuxiaobo@icdc.cn (X.L.); wuhaixia@icdc.cn (H.W.); 2 Ningbo Municipal Center for Disease Control and Prevention, Ningbo 315010, China; xugz@nbcdc.org.cn (G.X.); hetf@nbcdc.org.cn (T.H.); gushaohua1989@126.com (S.G.); 3 Guangdong Provincial Institute of Public Health, Guangdong Provincial Center for Disease Control and Prevention, Guangzhou 511430, China; mawj@gdiph.org.cn (W.M.); linhualiang2002@163.com (H.L.); gztt_2002@163.com (T.L.); 4 Beijing Center for Disease Control and Prevention, Beijing 100013, China; ZY968919@sina.com; 5 School of Population Health, University of Auckland, Private Bag 92019, Auckland 1010, New Zealand; a.woodward@auckland.ac.nz; 6 Environmental Change Department, Centre for Radiation, Chemical and Environmental Hazards, Public Health England, Chilton OX11 0RQ, UK; sotiris.vardoulakis@phe.gov.uk; 7 NIHR Health Protection Research Unit in Environmental Change and Health, London School of Hygiene and Tropical Medicine (LSHTM), 15-17 Tavistock Place, London WC1H 9SH, UK; sari.kovats@lshtm.ac.uk; 8 Public and Environmental Health Research Unit, London School of Hygiene and Tropical Medicine, 15-17 Tavistock Place, London WC1H 9SH, UK; paul.wilkinson@lshtm.ac.uk; 9 Department of Epidemiology, School of Public Health, Shandong University, Jinan 250012, China; 10 Changping District Centre for Disease Control and Prevention, Beijing 102200, China; lijing986169323@sina.cn

**Keywords:** cross-sectional survey, perception, greenhouse gas, mitigation, climate change, health co-benefits, China

## Abstract

Limited information is available on the perceptions of stakeholders concerning the health co-benefits of greenhouse gas (GHG) emission reductions. The purpose of this study was to investigate the perceptions of urban residents on the health co-benefits involving GHG abatement and related influencing factors in three cities in China. Beijing, Ningbo and Guangzhou were selected for this survey. Participants were recruited from randomly chosen committees, following quotas for gender and age in proportion to the respective population shares. Chi-square or Fisher’s exact tests were employed to examine the associations between socio-demographic variables and individuals’ perceptions of the health co-benefits related to GHG mitigation. Unconditional logistic regression analysis was performed to investigate the influencing factors of respondents’ awareness about the health co-benefits. A total of 1159 participants were included in the final analysis, of which 15.9% reported that they were familiar with the health co-benefits of GHG emission reductions. Those who were younger, more educated, with higher family income, and with registered urban residence, were more likely to be aware of health co-benefits. Age, attitudes toward air pollution and governmental efforts to improve air quality, suffering from respiratory diseases, and following low carbon lifestyles are significant predictors of respondents’ perceptions on the health co-benefits. These findings may not only provide information to policy-makers to develop and implement public welcome policies of GHG mitigation, but also help to bridge the gap between GHG mitigation measures and public engagement as well as willingness to change health-related behaviors.

## 1. Introduction

China is facing major challenges from both climate change and air pollution. Over the past 100 years, China has experienced noticeable climate changes, with the annual average air temperature increasing by 0.5–0.8 °C, a trend that is projected to intensify in the future [1]. The World Health Organization (WHO) has estimated, considering only the well understood impacts of dangerous climate change, and assuming continued progress in economic growth and health protection, that climate change is likely to cause approximately 250,000 additional deaths annually around the world between 2030 and 2050 [2]. According to the Global Climate Risk Index 2016, in 2014, China was identified as the 18th most affected country by weather-related extreme events among the total of 187 countries, and the ranking was 31th during the period 1995–2014 [3]. For air pollution, the Asian Development Bank reported that fewer than 1% of the 500 largest cities in China meet the air quality standards for PM_2.5_ recommended by the WHO, and seven Chinese cities are ranked among the 10 most polluted cities around the world [4]. In 2004, more than three-quarters of the Chinese urban population was exposed to air that did not meet the national air quality standards (GB3095-1996) [5]. Air pollution is the fourth leading risk factor for disease burden in China, leading to about 1.2 million premature deaths in 2010 [6].

In light of the situation and potential far-reaching health consequences and disease burden attributable to climate change and air pollution, further substantial actions are necessary to reduce greenhouse gas (GHG) and air pollutants emissions [7,8]. On 12 November, 2014, a joint China-US announcement vowed that China will stabilize its GHG emissions by 2030. The goal is “relatively ambitious”, since it means that at least 20% of China’s power will come from sources other than fossil fuels by 2030, up from around 10% in 2014, which could potentially reduce China’s gross domestic product (GDP) by 1% to 3.7% [9]. The reductions goal would make China’s agenda to mitigate air pollutants, GHG emissions and climate change more urgent and challenging. How to balance environmental and public health threats against near term economic prosperity may be the crucial challenge faced by developing countries like China.

**Figure 1:**
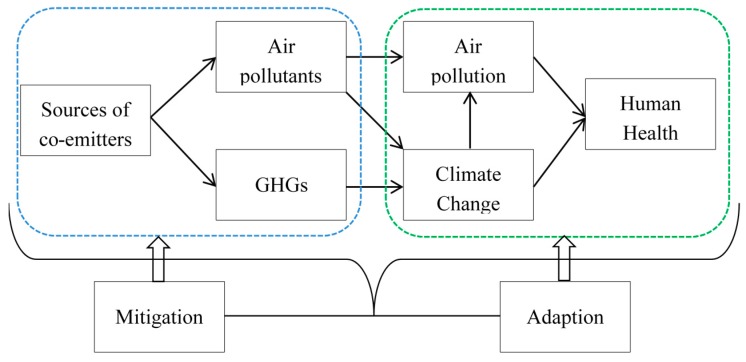
Pathway of the health co-benefits of greenhouse gas mitigation measures.

One way to help China address this challenge and achieve the climate change mitigation goal is to fully account for the health co-benefits of GHG emission reductions. According to the Fifth Assessment Report of the Intergovernmental Panel on Climate Change (IPCC), appropriate GHG mitigation measures aimed at curbing climate change will themselves have additional effects on public health, irrespective of the net effect on overall health gains and most of them beneficial [7,10]. One of the mechanisms for these so-called “co-benefits” is that GHGs and air pollutants to a large extent stem from the same sources and are linked in terms of their atmospheric formation and evolution as well as the effects on ecosystem and human beings [7,11,12]. Besides, some air pollutants such as black carbon and ozone are also climate-warming agents (namely greenhouse pollutants) with higher radiative forcing per unit than CO_2_ [12,13]. Thus, in addition to mitigating climate change, GHGs reductions may also deliver other improvements in public health simultaneously (Figure 1). For instance, GHG mitigation actions like reduced fossil-fuel combustion and improved energy efficiency can provide additional health benefits from reduced air pollution and related ill-health, especially in relation with the decrease in short-lived climate pollutants (e.g., black carbon and ozone), and the air quality benefits and health gains are often realized on a local scale and in the near-term [13,14,15]. These features of the ancillary health benefits may make GHG abatement measures much more attractive to local and national stakeholders (e.g., urban residents, key emitters and policy-makers), and can help motivate attempts and policies to put them into practice preferentially.

Although these health “co-benefits” of GHG mitigation strategies have been modeled or quantified in an increasing number of studies published in the scientific literature around the world in recent years [7,16,17,18], to date, no investigation has been conducted specifically to assess the perceptions of stakeholders about the ancillary health benefits of GHG emission reductions. In addition, despite the numerous studies focusing on the role of governments and economic sectors in curbing climate change, relatively limited attention has been paid to the contributions of individual factors (e.g., awareness, attitude and behavior) to GHG controls [8,19]. As the causes of climate change lie ultimately in human behavior, the collective effect of individual behavior change by many individuals may result in appreciable reductions in GHG emissions [7,17,20]. Engaging the public can also help generate support for effective climate change mitigation actions by businesses, and local and national governments [8,21]. A significant volume of studies have demonstrated the associations between perceptions and individuals’ willingness to change behavior and support for climate change mitigation, and mitigation policies may risk being ineffective or rejected when public lacking an understanding of the issue [21,22,23]. Besides, public health co-benefits of GHG abatement make impacts of climate change mitigation more geographically, temporally, and personally relevant and context-specific, which would be useful in encouraging public behavior change and support for climate policy. It is thus reasonable to assume that understanding the perceptions of stakeholders about the health co-benefits related to carbon emission reductions may contribute to the improvement in public engagement with mitigation measures, and addressing the problems of both climate change and air pollution as well as provide a driver for further mitigation efforts [8,24,25].

Aiming to fill this knowledge gap, we assess the perceptions of urban residents on the health co-benefits of GHG emission reductions in three cities in China. We firstly investigate the awareness level of the ancillary health benefits related with GHG abatement among subgroups within the survey population, taking into account demographic profiles. Then we analyse the perceptions of respondents about the health co-benefits in different economic or social sectors. Lastly, the potential influencing factors of respondents’ perception status are explored.

## 2. Materials and Methods

### 2.1. Study Design

A cross-sectional study “perceptive assessment of health risks caused by climate change, air pollution and health co-benefits of low carbon transition in China” was designed to investigate stakeholders’ knowledge of, attitudes toward, and perceptions of environmental issues in three Chinese cities. In this project, experts in the fields of climate change and air pollution, policy-makers, environmental scientists, and policy researchers collaborated closely. In order to achieve our objectives, literature review, existing policies analysis, workshops, questionnaire survey, and focus group discussions were conducted. As a part of this interdisciplinary project, the present study reports on a subset of the cross-sectional survey relating to questions on the health co-benefits of GHG emission reductions.

### 2.2. Study Settings

Three cities, Beijing, Ningbo and Guangzhou, were selected as the study settings, because they are representative of the different climatic zones and socio-economic areas in China. The three cities are typical of the Beijing-Tianjin-Hebei region (BTH), the Yangtze River Delta (YRD), and the Pearl River Delta (PRD), respectively, where there is a high degree of economic development, as well as high levels of air pollution and large carbon emissions [26]. Thus, information from these settings may mirror the current perceptions status of urban residents about climate change, air pollution and GHG emissions in China.

**Figure 2:**
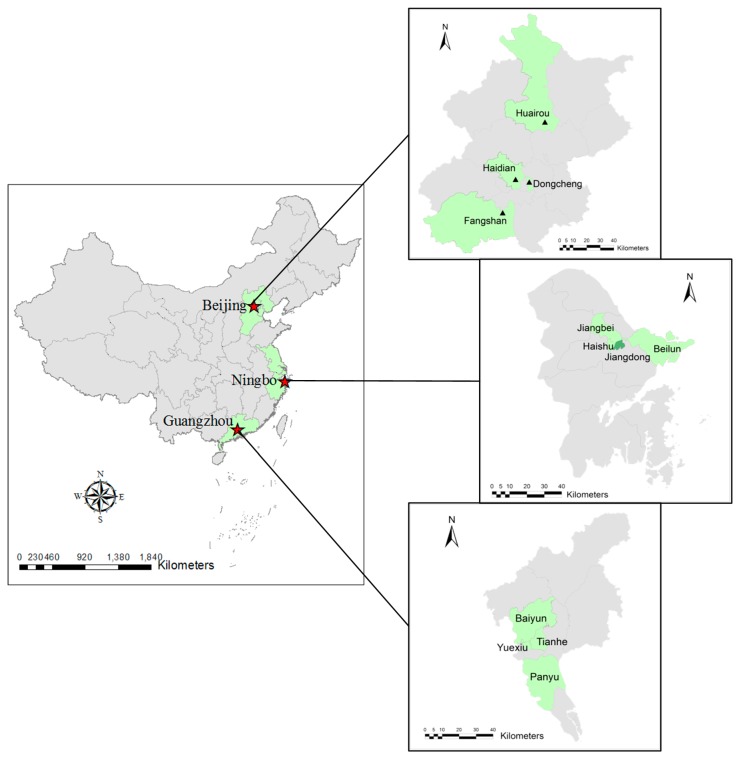
Study settings selected to conduct the field questionnaire survey.

With the hope of ensuring good geographical coverage and obtain information that best represents the diverse socio-economic and demographic characteristics of the local urban population in each city, four districts were targeted to perform the field questionnaire survey (Figure 2). The selection was based on multiple factors, including old and new districts, downtown and its surroundings, population size and density, as well as function and coverage of specific district (according to the newest statistical yearbook of each city). For example, in Beijing, there are four administrative divisions (Core Functional Area, Urban Function Extension Area, New Area of Urban Development, and Ecological Conservation Area) with different socio-demographic patterns, and we selected one district from each of the division (Figure 2). For each city, the survey was carried out in four Community Committees selected randomly from each district. The committees are usually located at the geographic centers of the specific communities and are well known among local residents.

### 2.3. Sampling and Participants

The sample size was estimated using Kish-Leslie formula for descriptive studies with single proportions [27]. There was no previous information on the level of people’s perceptions about the health co-benefits of GHG reductions. Hence, in order to obtain a relatively larger sample size, we hypothesized a prevalence of adult residents’ awareness of the ancillary benefits in each of the target city to be 30%. The sample size required, *n*, was generated as *n* = *pqz*^2^/*d*^2^, where, *p* was the assumed knowledge prevalence of the health co-benefits related to GHG abatement (*p* = 0.3); *q* = 1 − *p*; *z* was 1.96 (for 5% alpha error); and d was the level of precision (5%). This gave a required sample size of 323 participants, which was increased by 10% to compensate for potential non-respondents, resulting in the final study population of 360 for each city. The total required sample size for the three cities was 1080.

For each specific city, the pre-estimated sample size of 360 was stratified by gender and age groups (15–24, 25–44, 45–59 and over 60) representative of the distributions determined according to the latest statistical yearbook of each city, and then was proportionally distributed in each selected district. Participants were recruited purposely from the chosen committees, and the sample was based on quotas for gender and age in proportion to the population shares. Eligible respondents were permanent residents, aged over 14 years, who had lived in that community for at least six months. Only one eligible member was selected from each household to complete the survey. The final respondent sample from each city was compared to the characteristics of the corresponding target total population, and the population samples reflected similar gender balance and age distributions (Table 1).

### 2.4. Study Instrument

A semi-structured questionnaire was drafted following an extensive review of the scientific literature on health co-benefits in relation to GHG reductions. Discussions and deliberations among the researchers, and consultation with experts were also performed during the development of the preliminary questionnaire. An international workshop (attended by experts in the fields of climate change, air pollution, low carbon transition and policy research, public health officials and policy-makers from the three selected cities, interviewers, data collectors and administrators for the field survey) was organized to pre-test and review the draft questionnaire. The feedback and comments from attendees were then incorporated into the revised questionnaire. In order to assess the validity and reliability issues, before its use in the main investigation, the questionnaire was field-tested with a convenience sample of 21 urban residents from Changping district of Beijing. Based on the pilot study, participants’ comments on the content appropriateness, question clarity, and administration format were considered, and minor modifications were integrated to produce the final questionnaire and related interview guide.

The full questionnaire comprised the following five sections: Section “A”, included seven questions regarding awareness of health risks associated with climate change; Section “B”, included 10 questions about perceptions of health risks related to air pollution; Section “C”, included 18 questions regarding knowledge of, attitudes toward and perceptions of carbon emission reductions and the corresponding health co-benefits; Section “D”, included five questions about participants’ policy related concerns and recommendations. Socio-demographic information of the respondents was documented at the end of the interview with 12 questions (Section “E”). In total, the questionnaire contained 52 close-ended or open-ended questions. 3–5 point Likert-type items, i.e., 1 (Strongly Disagree), 2 (Disagree), 3 (Uncertain), 4 (Agree) to 5 (Strongly Agree), or other categorical items such as “Yes”, “No”, “Don’t know” or “Others” were employed to assess respondents’ knowledge of, attitude toward and perception of the mentioned environmental issues. This paper only reports on a subset of questions (Supplementary Materials), those related to health co-benefits of GHG abatement.

### 2.5. Data Collection

After the sampling protocol was developed, in order to ensure community support and engagement, the objectives and activities of the study were firstly delivered from our partners, the Centre for Disease Control and Prevention (CDC) of Beijing, Ningbo and Guangdong Province, to the selected District CDCs, then to the corresponding community health service centers (stations), and lastly to the randomly chosen Community Committees, and their participation were invited. All the selected committees showed their interest in and support of this study, and committed staff resources to the field investigation. 3–5 days before the surveys started, staff from each committee informed all permanent residents living in the community about the aims, contents, date and location of the survey, and encouraged them to participate. As an incentive to participate, all respondents were offered an approximately 30 Chinese Yuan (about 4 US dollars) gift (a laundry detergent sample) upon completion of their interview. Those who showed their willingness to take part in the interview then came to the committee to fill out the questionnaire, and participants were selected purposely according to the quotas for gender and age in proportion to the population shares.

In each city, before the field investigation, the questionnaires were administered firstly by interviewers involved in data collection to facilitate their understanding about the contents and questions. All interviewers received a half day intensive and systematic training. The training consisted of general information about the project, the purposes of the study, a review of the study’s interviewer manual, question-by-question review of the questionnaire, interview techniques and skills for approaching the participants, as well as social and cultural sensitivity issues during data collection, and an additional session for practicing the survey scripts with a partner. The first author and corresponding author reviewed each investigator’s initial interview, and pointed out inappropriate issues concerning the survey such as manners and choice of words.

From October to November of 2015, participants were interviewed by the trained investigators using the semi-structured questionnaire. In each site, one to four senior researchers were always present in the field to monitor the process, coordinate the questionnaire collection and, examine the quality of the data collected. The staff involved were familiar with the survey procedure documents, and communicated with the first author or corresponding author for any questions and clarification when needed. All completed questionnaires were reviewed and checked for completeness and validity by the field supervisors (senior researchers). Incomplete questionnaires were returned to the respondents immediately to figure out the potential reasons (e.g., difficulties in understanding the questions, omission, refuse to cooperate, or unexpected exigency), and the completion was asked for if possible (in the case of permission and cooperation). The reasons found out (with the help of the staff from the local community health service centers or Community Committees) will be delivered to the investigators as feedback in order to prepare for or avoid similar situations. The survey in each site was carried out continuously until the required sample size was met.

### 2.6. Statistical Analysis

The collected data were double entered into a database by trained personnel using EpiData 3.1 software (EpiData Association, Odense, Denmark). The data were then transferred to statistical software and analyzed according to different variables. Firstly, descriptive statistics were used to illustrate demographic characteristics of participants and percentages of categorical variables. Secondly, a series of Chi-square tests were employed when appropriate to examine the associations between socio-demographic variables and perceptions of participants on the health co-benefits of GHG mitigation (1 = never heard, 2 = only heard, and 3 = familiar); otherwise, Fisher’s exact test (expected cell frequencies less than or equal to five) was used. Thirdly, the distributions and percentages of perception variables in different economic or social sectors were summarized. The primary purpose for this series of questions was to further investigate the awareness of respondents on how GHG reductions can bring about health co-benefits, as well as re-check the perceptions of participants about the contents of the health co-benefits.

In addition, unconditional logistic regression analysis was performed to assess the associations of demographic variables, attitude (from 1 = Strongly Disagree, to 5 = Strongly Agree) and practice factors involving environmental issues (independent variables) with perceptions of respondents on the health co-benefits related to GHG abatement (dependent variable, coded as 0 = not familiar, and 1 = familiar). To determine which factors were associated with the perception variable, logistic regression analysis was done at both bivariable and multivariable level. Factors that had a *p*-value < 0.05 and those identified in the literature review to have potential effects on the perceptions of co-benefits were included in the final multivariable model, and the odds ratios (OR) with 95% confidence intervals (CI) were calculated. All statistical analyses were performed using IBM SPSS 19.0 (SPSS Inc., Chicago, IL, USA) and Stata 12.1 (Stata Corporation, College Station, TX, USA). All statistical tests were two-sided and a *p* value less than 0.05 was considered to be statistically significant.

### 2.7. Ethical Statement

Ethical approval was granted by the Ethics Committee of the Chinese Center for Disease Control and Prevention (No. ICDC-2015005). The survey was anonymous, and verbal informed consent was obtained from all participants prior to each interview, after explaining the objectives of the study. Respondents were assured that the privacy and confidentiality of the data was to be maintained. Sufficient time was given to participants to complete the questionnaire, and it was emphasized that the participation was voluntary and they had the right to refuse participation or withdraw from the survey at any time.

## 3. Results

### 3.1. Socio-Demographic Characteristics

A total of 1166 participants took part in the survey, but seven respondents did not complete the survey after having started it, because of difficulties in understanding the questions, distractions, or unexpected exigency, as reported by the interviewers, so finally 1159 completed questionnaires were included in this analysis, representing an overall response rate and survey completion rate of 99.4%.

Table 2 presents the demographic characteristics of the total sample. Of the 1159 participants, individuals aged from 25 to 59 years were the majority (66.7%), and women accounted for 51.3%. Ethnically, 97.4% of the participants were Han Chinese, similar to the actual ethnicity distributions in each city. The sample covered various education levels and occupations, and more than half of the respondents (55.1%) with family monthly average income between 2000 and 5000 Chinese Yuan (about 290–725 US dollars, and the national monthly per capita disposable income of urban households in 2015 was 2649.17 Yuan). Participants from urban and rural (according to registered residence, namely Hukou) accounted for 75.5% and 24.5%, respectively.

### 3.2. Awareness on the Health Co-Benefits of GHG Reductions

Participants were asked about their perceptions of the health co-benefits in relation to GHG emission reductions. We found widespread agreement (91.9% participants) that GHG abatement do not only mitigate climate warming, but also improve public health in various ways. Table 3 shows the responses of participants regarding the health co-benefits of GHG reductions. Despite the high recognition of the ancillary health benefits relating to GHG mitigation, relatively few (15.9%) respondents reported that they were familiar with the specific types of health co-benefits, while the majority (67.8%) admitted that they had only heard about the concept but could not figure out the details. Although all three cities presented low awareness level about the ancillary health benefits, participants from Ningbo showed a statistically significant (*p* = 0.002) higher perceptions level (21.4%) than in the other two cities.

**Figure 3:**
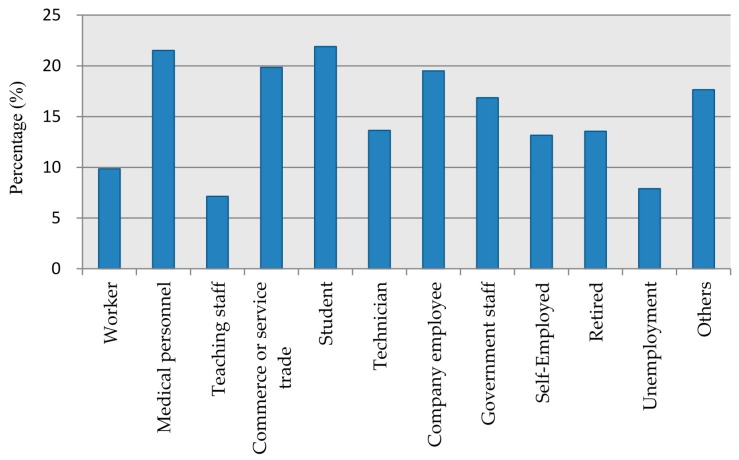
Percentage of respondents who self-reported that they were aware of the health co-benefits in relation to GHG reductions among different occupations.

The levels of awareness of the health co-benefits varied significantly across different age groups of respondents (*χ*^2^ = 59.94, *p* < 0.001). In general, younger participants tended to have higher awareness level. The gender of respondents did not seem to play a role in the level of awareness of health co-benefits (*p* = 0.25). There was an association between individual education and the belief that GHG reductions would bring improvement in public health (*χ*^2^ = 91.67, *p* < 0.001), as respondents with higher education level tended to be more aware of the ancillary health benefits of GHG mitigation. Family income and registered residence played a statistically significant role as well. The perceptions of participants increased with family income, except for the group of <1000 Chinese Yuan (*χ*^2^ = 22.79, *p* = 0.012). While 17.0% of respondents from urban areas reported they were aware of the health co-benefits, fewer participants (12.3%) from rural areas claimed to be familiar with the concept (*χ*^2^ = 6.26, *p* = 0.044). Respondents’ marital status seemed to be associated with the level of awareness of health co-benefits (*χ*^2^ = 14.56, *p* = 0.017), but the factor of participants’ health status did not appear to have an effect. Figure 3 shows the distribution of the respondents who were familiar with the health co-benefits by occupation. Among all participants, students, medical personnel, staff of commerce or service trade and company employees presented relatively higher proportion in the awareness of the health co-benefits.

### 3.3. Perceptions on the Ways of GHG Mitigation to Improve Public Health

**Figure 4:**
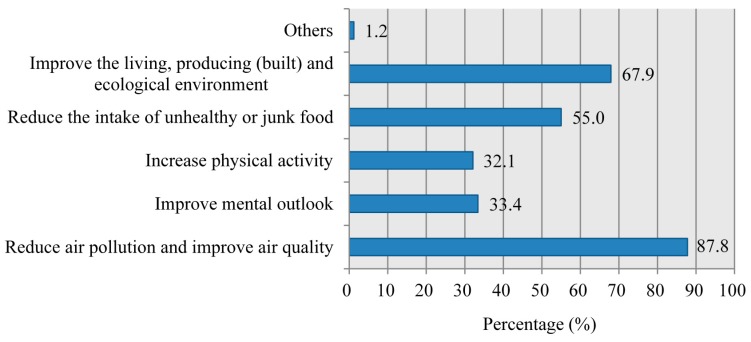
Perceptions of respondents on the pathways of GHG emission reductions to provide ancillary public health benefits.

Participants were asked about the potential pathways through which carbon emission reductions can bring about additional health gains. As shown in Figure 4, a large majority of respondents (87.8%) indicated that GHG mitigation measures can reduce indoor air pollution and improve outdoor air quality, and consequently protect public health from the hazards of air pollution. Roughly two thirds (67.9%) reported that the low carbon transition could improve living, producing (built) and ecological environment, which improves human health and wellbeing. This could be achieved by “increasing the amount of physical activities” and “reducing the intake of unhealthy or junk food (e.g., food with high fat content)” according to 32.1% and 55.0% of the respondents, respectively. Of special note is that 33.4% of the participants indicated that a low carbon lifestyle can improve their mental outlook.

### 3.4. Perceptions on the Health Co-Benefits in Different Sectors

Table 4 summarizes the perceptions of respondents on the health co-benefits of GHG mitigation in different economic or social sectors. Participants generally showed high levels of awareness, but with some confusion to different degrees. For instance, to the question “In energy production and use, through what ways could carbon emission reductions bring about health co-benefits?” Almost all respondents (95.9%) selected “decrease air pollutants, improve air quality, and reduce diseases caused by air pollution”; and “mitigate climate change, decrease the burden of climate-sensitive diseases” was selected by 88.6% of the respondents. However, “increase physical activity, reduce obesity and cardiovascular diseases” and “encourage scientific innovation and facilitate social development” which are exactly not the pathways that GHG mitigation policies bring about health co-benefits in energy generation, were also indicated by 75.8% and 73.8% respondents, respectively. Similar relatively high perception levels, along with some misunderstanding about the pathways of GHG abatement measures that can generate health co-benefits, were also observed in the transport, agriculture and household sector (Table 4).

### 3.5. Predictors of Respondents Perceptions on the Health Co-Benefits

A series of bivariate logistic regression analyses were conducted firstly to examine the associations between the dependent variable (perceptions of the health co-benefits) and each independent variable. After the variables filter, factors with statistically significant impacts on the dependent variable were included in the final multivariable logistic model, including “Age”, “Education level”, “Attitudes toward the current urban air pollution”, “Attitudes toward governmental policy attempts and progress to deal with the problems of air pollution and climate change”, “Have respiratory diseases”, and “Choose low carbon lifestyle in daily life or work”. The only exceptions were two variables (“Gender” and “Family monthly average income”), which did not have significant bivariate associations with the perception variable but were still included in the final model, since they were plausible and were considered a priori to be important variables.

Table 5 presented the final logistic regression analysis for the associations between independent variables and respondents’ perceptions on the health co-benefits of GHG reductions. The age of participants played a negative role in awareness of individuals about the health co-benefits (OR = 0.98, 95% CI: 0.97–0.99). By contrast, these four variables—attitudes of participants toward air pollution and governmental policy efforts, suffering from respiratory diseases, or following low carbon lifestyle in usual—exerted significant positive influence on respondents’ perceptions about the ancillary health benefits. The effect of gender, education level and family income on the perceptions of respondents appeared statistically non-significant (*p* > 0.05).

## 4. Discussion

Given the role of the so-called “health co-benefits” in motivating emitters to put GHG mitigation measures into practice at individual, social, national and even global level, the potential associations between perception of the ancillary health benefits and individuals’ willingness to change behavior and support for low carbon transition, and the challenges of air pollution and climate change faced China, it is important to assess the perceptions of the health co-benefits of GHG emission reductions among stakeholders in different regions in China. In this study, the perceptions of urban residents on the health co-benefits of GHG mitigation, and the relevant influencing factors were investigated. To our knowledge, this is the first study to specially assess the awareness of the health co-benefits in relation to GHG emission reductions around the world. Therefore, findings from the study have the potential to fill an important knowledge gap.

Evidence from health belief models, risk perception attitude frameworks and social cognitive theory have proposed significant, although sometimes small, associations between knowledge of the issue, risk perceptions and the likelihood of actions [21,28,29]. If properly executed, under specific conditions, and with certain target audiences, information may lead to increased awareness and this may lead to behavior changes [30]. For example, studies have shown that response of population to natural hazard warnings is partly determined by perceived urgency of the threat [31], and the risk perceptions need to be accompanied by efficacy beliefs and proximity to the hazard to promote action [21,28]. However, for climate change, individual perception of risk is a necessary but far from sufficient factor on its own, to contribute to motivating individuals to change their behavior [22]. Instead, it is emotions—the feelings along with thinking—that are central [32]. It should be noted that negative emotions such as fear, guilt and pessimism, are likely to produce passive and defensive responses, and hardly do much to encourage people to change their behaviors or to press for wider social action [8]. Several studies have focused on risk perceptions of climate change, but less attention has been paid to the awareness of the health benefits associated with mitigation actions [33,34]. In addition to providing engaging messages about how to address the problem, ancillary health gains of GHG reductions may also represent climate change mitigation in ways that connect with people’s core ideologies and identities and then anchor it in positive emotions, which is one of the crucial determinants of behavior and behavioral change [8,35,36]. Besides, the ancillary health benefits make GHG abatement measures more “down to earth” and personally-relevant issues, which is an evolutionary tendency for people to pay attention to and appears to be particularly compelling [24,37,38]. Reframing climate change from an environmental to a public health issue and linking GHG mitigation policies to beneficial health gains (positive vision) may bring climate change mitigation closer to home thereby increasing its relevance to the public, and potentially encouraging public engagement in mitigative behavior change [25,37,39]. Thus, this perceptive assessment of the health co-benefits in relation to GHG abatement may fill some of the evidence gaps and provide additional motivation to help change individuals’ climate-affecting behaviors and habits.

Similar with previous research on risk perceptions of climate change [25,33,34], we found that awareness of the multiple benefits of GHG emission controls on climate and public health was very high (91.9%) in the three cities of this study, likely due to the extensive media coverage, internet penetration and government advocacy on this topic in China. In recent years, awareness raising of low carbon development and green growth has been carried out continuously across the whole country, and it seems that the idea of low carbon transition has been absorbed by the general public [26]. This information increase is reflected in growing numbers of scientific reports, newspaper articles, and increased awareness of and interest in international negotiations devoted to the issue. Though the knowledge was widespread, (more concrete) awareness of the health co-benefits of GHG reductions was less common, only 15.9% respondents claimed they were familiar with the specific concept. When the three cities were compared, despite some statistically significant differences between cities, we found large proportions of the study populations with low awareness level of the health co-benefits, which indicates that further public health outreach campaigns on the topic of the health co-benefits of GHG mitigation are needed.

Results showed that young participants tended to show higher recognition of the health co-benefits, and this relationship was also observed in the final multivariable logistic model. The reasons for this may be that the health co-benefits of GHG mitigation is a relatively new concept raised in recent years [7,17], and young individuals usually have the advantage of learning and absorbing emerging information through more channels and faster than older individuals [40]. For example, young individuals may access information through channels such as school education, the internet, smart phone, television, radio, and newspaper, while older individuals mostly obtain information through only traditional media like television and newspaper [25,40]. According to China Internet Network Information Center, new media based on internet and smart phones are becoming a key pathway to spread information in China, with 74.7% of the internet users aged 10–39 years [41]. Besides, the younger age groups are reportedly more likely to be concerned about environmental issues such as air pollution and climate change [25,42]. Currently, no studies have assessed gender differences with respect to the perceptions of health co-benefits relating to GHG reductions. While evidence from risk perceptions assessment of climate change or heatwave is still not uniform, women seem to be generally more concerned about environmental issues and are aware of environmental risks than men [25,40,43,44]. In our study, although women tended to demonstrate higher awareness level of the health co-benefits, the difference between genders was statistically non-significant (*p* = 0.192).

We found a relationship between education and belief that carbon emission reductions could generate health co-benefits; higher education levels were associated with better awareness. Education is associated also with a greater probability of expressing concerns about environmental threats such as climate change, heatwaves and air pollution [25,45,46]. It has been suggested that modern lifestyles largely disconnect individuals from directly experiencing changes and instead make them more dependent on mediated information about environmental issues (e.g., internet, smart phones or television) [22,47]. It was reported that compared with those with lower education levels, individuals with higher education may be more willing to search health-related information, and more likely to access climate change information through the internet [34,43]. However, the education variable did not have a significant effect in the final logistic model (*p* = 0.757), partly due to the adjustment for age, gender and income, which were often interlinked and overlapping with education [40]. Respondents with high household income were more likely to report awareness of the health co-benefits. Similarly, individuals with urban registered residence were more aware of the benefits of GHG reductions. Compared with rural participants, urban respondents were relatively higher-income earners who could access information through more channels like internet, smart phones, television or newspapers [25,40,41]. Higher income is often associated with higher education levels, while is predicted to make people more aware of environmental issues [48,49]. Besides, it was suggested that there was a higher probability for urban residents to be concerned about climate change than their rural counterparts [45].

Also as expected, respondents who are students, medical personnel, staff of commerce or service trade and company employees were more likely to be aware of the health co-benefits, which could be explained by the fact that they were mostly younger individuals who are the main internet users in China [41]. This may also explain the different awareness levels among participants with different marital status. It is not surprising perhaps that, medical personnel who are trained in public health were more likely to be interested in environment-related information [34,43]. Surprisingly, teaching staff and technicians showed relatively lower awareness levels, implying further research is needed to establish whether this is related to lack of information or personal motivation that may have led to the lower awareness levels. For the perceptions on the ways GHG mitigation can improve public health, although the majority of respondents recognized reducing air pollution and improving the environment as ways of carbon emission reductions that can bring additional health gains, relatively few individuals indicated “increase physical activity” or “reduce the intake of unhealthy or junk food” as the potential approaches. 33.4% participants insisted that GHG abatement measures can improve people’s mental outlook, suggesting the additional value of relevant low carbon measures (e.g., sustainable urban design and urban green space) in the improvement of public emotional and mental health, such as enhancing cultural and aesthetic values, increasing social contact and neighborhood cohesion, and offering restorative experiences [8,15,18,38,39]. However, in terms of the relatively low awareness level, these aspects require more investigation and health education campaigns.

Overall there was close to consensus about the statements on the health co-benefits of GHG mitigation in different economic or social sectors (Table 4). Plausible explanations for this phenomenon are summarized as following. First, widespread awareness of the risks of climate change or temperature warming have been observed by numerous studies around the world [28,33,34,40,43,49,50]. As a similar environmental issue, and in terms of the increasing governmental and social media awareness raising activities in recent years in China, widespread approval of the statements of the health co-benefits related to GHG reductions may be expected; Second, though there is a substantial awareness, it is likely that there is some extent of confusion or misunderstanding among the respondents in terms of differentiating the concept of “health co-benefits” from “social benefits or welfare benefits”; Third, social desirability may be a factor influencing responses to the survey. As an emerging concept, when participants know little or understanding not well about the health co-benefits of GHG abatement, then a convenient answer may be provided. Or individuals may have been worried about giving “wrong” answers, and the socially desirable ones were chosen [51]. For instance, in the context of the increasing governmental and social policy advocacy and publicity of low carbon transition and green growth in recent years, various positive effects of GHG reductions have been gradually becoming a socially-acceptable consensus in China, especially in regions that the most economically developed while undergoing the most serious air pollution and largest carbon emissions, such as Beijing, Ningbo and Guangzhou. Thus, when some participants from the three studied cities do not understanding the specific content of the health co-benefits well, they may be to relate some positive outcomes (especially outcomes with the words like “improvement”, “development”, “innovation”, or “decrease”) to the impacts of GHG abatement measures according to social expectations (courtesy bias). In light of the aborative development of the study instrument (questionnaire) and quality control of survey process, another less possible reason may be that too many messages were delivered or the questionnaire questions were too complicated. To save time, some respondents might have only absorbed the simplest of these pieces of the questions, and checked similar answers for each question rather than spending the necessary time to think carefully about each of them [42,49].

Participants who agreed the air pollution in their city was a serious problem and has caused health impacts, or approved that government has taken a package of measures to address climate change, air pollution and carbon emissions and the situations is improving, were more likely to be aware of the health co-benefits of GHG mitigation. Consistent with the discussion above, a higher level of concern about environmental issues such as climate change, air pollution or carbon emissions is more likely to be associated with the awareness of the health co-benefits. Similarly, respondents with respiratory diseases may be more concerned about air pollution and relevant mitigation measures, which increases their chances to access the information involving the health co-benefits of GHG reductions, making this variable a significant predictor of the perceptions status (OR = 1.50, 95% CI: 1.01–2.25). The final logistic regression model also revealed that the individuals who followed low carbon lifestyles tended to be more aware of the health co-benefits. This association may be explained by a low carbon lifestyle increasing their chances to learn about the health co-benefits, or by the recognition of the health co-benefits in relation to GHG mitigation actions motivating them to follow a low carbon lifestyle.

### Strengths and Limitations

The present study has several strengths. Firstly, to the best of our knowledge, it is the first study to assess specially the perceptions of urban residents on the health co-benefits in relation to GHG reductions around the world. Findings from this study, together with other research initiatives currently under way in the UK-China research project “China Prosperity Strategic Programme (SPF 2015-16)” (perceptive assessment of health risks caused by climate change, air pollution and health co-benefits of low carbon transition in China), will, it is hoped, fill some of the knowledge gaps on the topic of GHG mitigation. Secondly, the three studied cities were selected from the BTH, YRD and PRD regions, which are typical of three different climatic zones in China, where the socio-economic fields achieve the greatest success while undergoing the most serious air pollution and facing greatest challenge of carbon emissions in China. Thus, findings based on these cities may are representative of the perception status of urban residents about the health co-benefits of GHG abatement in most developed parts of China. Thirdly, our field survey involving three cities and twelve districts, activities such as objective publicity, local investigators training and focus groups during the surveys, may play a role in improving the understanding of the health co-benefits of low carbon transition among local experts, CDC officials, staff of each committee, and interviewers. Improved perceptions making it is possible for the stakeholders to take the health co-benefits into consideration in their daily life, routine activities or institutional operations, which ultimately help to facilitate the development and implementation of low carbon policies in China. For example, during the focus group discussions, after discussing and understanding the health co-benefits of mitigation measures in different economic sectors, most participants claimed that given the individually and environmentally beneficial gains (personally-relevant health benefits and air quality improvement) of GHG reductions, they would like to consider alternative options (low carbon and green) in their routines and habits, such as choosing active travel (walking, cycling, and public transport) for short city trips, limiting or changing westernized dietary patterns that mainly from animal sources and rich in saturated fat and sugar, and shifting carbon-dependent consumption customs to environmentally-friendly lifestyles. In general, respondents regarded these low-carbon alternative options as mitigation actions that could be realistically achieved at the local and personal level (details will be discussed in a forthcoming companion paper). Fourthly, our survey revealed that the awareness levels of respondents about the health co-benefits of carbon emission reductions are very low (15.9%), while certain segments of the population were more likely to be aware of the health co-benefits than others, such as those who were younger, more educated, with higher family mean income, and more concerned about air pollution, climate change and government efforts. These findings could provide helpful information to policy-makers to develop and implement win-win policies for air pollution and climate change mitigation. They may also improve public acceptability of GHG mitigation measures, and the necessity to conduct extensive GHG reductions-related health education campaigns, at both national and local level. Besides, in light of the appreciable health co-benefits of GHG abatement, our findings also hold the potential to help bridge the gap between present and future GHG abatement measures and public support and engagement, as well as the willingness to change their behaviors. For instance, although public health co-benefits of GHG mitigation are plausible and attractive to those people interviewed, participants indicated that their behavior change was often constrained by the lack of enabling infrastructures and mechanisms. To be specific, some respondents pointed to a lack of acceptable and reliable built environment (e.g., urban green space) and public transport in their locality for active travel, unaffordable low carbon goods and household appliances, and intractable social norms and expectations that requiring carbon-dependent consumption customs and lifestyles (e.g., animal sources foods, private motor vehicles, and high-emission electronic goods). This information may inspire governments to undertake further practice change and actions to reduce or remove the barriers gradually, through health education campaigns creating environmental citizenship, in combination with a framework of incentives and regulations (Carrots and Sticks) [38,39] (details will be presented in a forthcoming companion paper).

Several limitations of this study should also be acknowledged. First, the cross-sectional nature of this study does not allow for the assessment of trajectories [44,51]. By relying on survey-based evidence from a single point in time, we are unable to uncover the causal relationships between independent variables and the perceptions of respondents on the health co-benefits of GHG mitigation. For instance, in the final multivariate regression model, we cannot establish whether it is the low carbon lifestyle influencing the awareness of respondents, or individuals’ perceptions of the health co-benefits lead to low carbon lifestyles. Second, the study was conducted on a convenience basis, did not involve a random sample (selected purposely). Hence this study provides only a snapshot of urban residents’ perceptions on the health co-benefits, the results may not representative of the entire population, and are not necessarily generalizable to other regions. However, there were four districts with different socio-economic features in each city were investigated, and our findings are based upon a sample of sufficient size and with similar socio-demographic characteristics of the target population of each city. Thus, a reasonable reflection of the awareness of urban residents on the health co-benefits of GHG reductions in the three studied cities could be provided by this survey. Third, all information about the perceptions of the health co-benefits are based on respondents’ self-reports, which is, by definition, subjective. As with all studies of this kind, the answers may be affected by the biases of social desirability and courtesy, meaning that participants are prone to report the answers that they perceive to be socially acceptable, or that they thought the interviewer wanted to hear and in the favor of the survey, which may be different from their real perceptions [21,33,40,41,52]. However, a series of measures including but not restricted to investigator training, explanation of survey objectives, anonymity of the survey, and assurance of privacy were taken to deal with these potential biases. Fourth, the present survey focuses on the permanent residents in the three cities, newcomers from other regions (less than 6 months) were not included, so our results cannot be extrapolated to these population sub-groups. Another limitation of this study pertains to the fact that the shortage of similar studies carried out in China or other regions of the world makes the comparison and interpretation difficult. Therefore, information from surveys about risk perceptions of climate change or heatwaves conducted around the world were borrowed as references [21,22,25,33,34,40,42,43,44].

## 5. Conclusions

The perceptive assessment of the health co-benefits of GHG emission reductions carried out in this study sheds some lights on the existing knowledge gaps. Overall, individuals’ awareness of the health co-benefits of GHG reductions is still limited, as only 15.9% of the participants stated that they were familiar with the specific contents of the health co-benefits. Considering the potential confusion or misunderstanding among the respondents about the “health co-benefits” versus the “social benefits or welfare benefits” presented in Table 4, the actual awareness level of participants may be even lower. Perceptions of the health co-benefits related to GHG mitigation are influenced by socio-demographic characteristics of participants; those who are younger, more educated, with higher family average income, and with urban registered residence, were more likely to be aware of the ancillary health benefits than others. The final logistic regression model revealed that age, attitudes toward the air pollution and governmental efforts to address the problem, suffering from respiratory diseases, and following low carbon lifestyle in daily life or work, were significant predictors of respondents’ awareness about the health co-benefits of GHG abatement.

The influencing factors of respondents’ perceptions on the health co-benefits identified in this study, may help bridge the gap between GHG mitigation measures and individuals’ knowledge of, attitudes toward and perceptions of carbon emission reductions. With the advance of the low carbon transition in China, which is likely to encounter resistance from parts of the society, the health co-benefits of GHG emission controls will continue to be an important driver for strategies aiming to remove barriers to mitigation in the context of both climate change and air pollution. Acknowledging insufficiency in both the scientific information production and public interpretations of the health co-benefits of GHG reductions is a necessary step in improving public awareness, follow and support of GHG abatement measures in China. This compliments moves towards a more comprehensive framework for public education campaigns explaining the health co-benefits of carbon emission reductions, accompanied by well-resourced and sustained policies enforcement, which could stimulate more productive engagement between scientific knowledge, individuals’ perceptions and changes in behaviors [8,24]. Individuals are the actors who ultimately initiate, inspire, guide and enact the reductions in GHG emissions to curb climate change [22], and public perceptions may be of considerable interest to policy-makers, as they can drive policy as much as scientific risk assessments [44,53]. Therefore, the public may be the strongest ally in the battle against carbon emissions. In order to let the health co-benefits of GHG reductions become a mobilising, engaging and effective instrument in regional or even global health thinking, the participation, commitment and ownership of individuals (“grassroots”) is needed [7,8,54].

Our findings demonstrate that people’s perceptions and the influencing factors are complex and are often difficult to predict, pointing to the value of further work on public understanding of the health co-benefits of GHG emission reductions in China. Public health awareness campaigns and information dissemination are important to deliver the health co-benefits, but need to be accompanied by careful inquiry into attitudes on the merits of emission controls and behavioral change towards low carbon lifestyles [8,26]. More research is needed to understand which communications methods are the most effective to make this happen. In this context, the call for “more information” means that attractive and public acceptable scientific knowledge with the potential to motivate people to engage in behaviors that reduce GHG emissions is needed, rather than just evidence filling an “information-deficit” related to the health co-benefits of climate change mitigation. Of special note that it is often the “no concern” or “value-action gap” that impede public participation and action, which may eventually undermine policy implementation of low carbon transition [7,8,26,55], requiring further awareness raising and behavioral research.

## Figures and Tables

**Table 1:** Demographic information of the sample versus the target total population of each studied city.

Items	Study Participants (*N*, %)	Target Population (%)
**Beijing**	369	21.15 million (in 2013)
Age		
15–24	64 (17.3%)	18.3%
25–44	151 (40.9%)	44.2%
45–59	91 (24.7%)	22.2%
≥60	63 (17.1%)	15.3%
Gender		
Male	179 (48.5%)	50.1%
Female	190 (51.5%)	49.9%
**Ningbo**	373	7.66 million (in 2015)
Age		
15–24	49 (13.1%)	16.8%
25–44	148 (39.7%)	39.3%
45–59	114 (30.6%)	27.2%
≥60	62 (16.6%)	16.7%
Gender		
Male	184 (49.3%)	50.9%
Female	189 (50.7%)	49.1%
**Guangzhou**	417	12.87 million (in 2014)
Age		
15–24	100 (24.0%)	25.2%
25–44	196 (47.0%)	45.1%
45–59	73 (17.5%)	18.7%
≥60	48 (11.5%)	11.0%
Gender		
Male	202 (48.4%)	51.9%
Female	215 (51.6%)	48.1%

**Table 2:** Demographic characteristics of the study participants (*N* = 1159).

Characteristic	Category	Number (*N*)	Percent (%)
Age (years)	15–24	213	18.4
	25–44	495	42.7
	45–59	278	24.0
	≥60	173	14.9
Gender	Male	565	48.7
	Female	594	51.3
Ethnic group	Han	1129	97.4
	Others	30	2.6
Education level	Primary school or below	72	6.2
	Junior middle school	226	19.5
	Senior middle/vocational school	333	28.7
	Bachelor degree	481	41.5
	Master degree or above	47	4.1
Marital status	Unmarried	277	23.9
	Married	844	72.8
	Widowed	23	2.0
	Divorced	15	1.3
Occupation	Worker	132	11.4
	Medical personnel	79	6.8
	Teaching staff	42	3.6
	Commerce or service trade	126	10.9
	Student	105	9.1
	Technician	22	1.9
	Company employee	164	14.2
	Government staff	89	7.7
	Self-Employed	114	9.8
	Retired	214	18.5
	Others	72	6.1
Family monthly income per person (Chinese Yuan, 2015 national average is 2649.17 Yuan)	<1000	38	3.3
	1000–2000	102	8.8
	2000–3000	299	25.8
	3000–5000	340	29.3
	5000–10,000	255	22.0
	>10,000	125	10.8
Registered residence (Hukou)	Urban	875	75.5
	Rural	284	24.5

**Table 3:** Perceptions of respondents (by total and subgroups) on the health co-benefits of GHG emission reductions.

Variables	Perceptions of the Health Co-Benefits (*N*, %)				
	Never Heard	Only Heard	Familiar	*χ*^2^	*p*
Total	189 (16.3)	786 (67.8)	184 (15.9)		
City					
Beijing	54 (14.6)	268 (72.6)	47 (12.8)	16.56	0.002
Ningbo	67 (18.0)	226 (60.6)	80 (21.4)		
Guangzhou	68 (16.3)	292 (70.0)	57 (13.7)		
Age (years)					
15–24	27 (12.7)	148 (69.5)	38 (17.8)	59.94	<0.001
25–44	47 (9.5)	358 (72.3)	90 (18.2)		
45–59	66 (23.7)	176 (63.3)	36 (13.0)		
≥60	49 (28.3)	104 (60.1)	20 (11.6)		
Gender					
Male	99 (17.5)	370 (65.5)	96 (17.0)	2.75	0.25
Female	90 (15.2)	416 (70.0)	88 (14.8)		
Education level					
Primary school or below	36 (50.0)	33 (45.8)	3 (4.2)	91.67	<0.001
Junior middle school	51 (22.6)	137 (60.6)	38 (16.8)		
Senior middle or vocational school	48 (14.4)	235 (70.6)	50 (15.0)		
Bachelor degree	43 (8.9)	354 (73.6)	84 (17.5)		
Master degree or above	11 (23.4)	27 (57.5)	9 (19.1)		
Marital status					
Unmarried	43 (15.5)	185 (66.8)	49 (17.7)	14.56	0.017
Married	132 (15.6)	582 (69.0)	130 (15.4)		
Widowed	11 (47.8)	10 (43.5)	2 (8.7)		
Divorced	3 (20.0)	9 (60.0)	3 (20.0)		
Family monthly income per person (Chinese Yuan)					
<1000	7 (18.4)	24 (63.2)	7 (18.4)	22.79	0.012
1000–2000	20 (19.6)	69 (67.7)	13 (12.7)		
2000–3000	52 (17.4)	205 (68.6)	42 (14.0)		
3000–5000	70 (20.6)	219 (64.4)	51 (15.0)		
5000–10,000	24 (9.4)	190 (74.5)	41 (16.1)		
>10,000	16 (12.8)	79 (63.2)	30 (24.0)		
Registered residence (Hukou)					
Urban	132 (15.1)	594 (67.9)	149 (17.0)	6.26	0.044
Rural	57 (20.1)	192 (67.6)	35 (12.3)		
Health status					
Poor	9 (22.0)	26 (63.4)	6 (14.6)	6.57	0.16
Average	61 (14.9)	294 (72.1)	53 (13.0)		
Good	119 (16.8)	466 (65.6)	125 (17.6)		

**Table 4:** Perceptions of respondents on the health co-benefits in relation to GHG mitigation measures in different economic or social sectors.

Statements	Agree	Disagree	Uncertain
	*N* (%)	*N* (%)	*N* (%)
**Health co-benefits of carbon emission reductions in the energy sector**			
Increase physical activity, reduce obesity and cardiovascular diseases	879 (75.8)	115 (9.9)	165 (14.3)
Mitigate climate change, decrease the burden of climate-sensitive diseases	1027 (88.6)	52 (4.5)	80 (6.9)
Encourage scientific innovation and facilitate social development	855 (73.8)	102 (8.8)	202 (17.4)
Decrease air pollutants, improve air quality, and reduce diseases caused by air pollution	1112 (95.9)	14 (1.2)	33 (2.9)
**Health co-benefits of carbon emission reductions in the transport system**			
Improve the quality of vehicles and decrease road traffic injuries	754 (65.0)	205 (17.7)	200 (17.3)
Promote the development and use of low carbon and environmental friendly vehicles	1079 (93.1)	31 (2.7)	49 (4.2)
Improve physical activities, decrease cardiovascular diseases, obesity and diabetes through promoting active travel (walking, cycling and public transport)	1008 (87.0)	58 (5.0)	93 (8.0)
Decrease vehicle use and air pollutants emission, improve air quality and public health	1109 (95.7)	15 (1.3)	35 (3.0)
**Health co-benefits of carbon emission reductions in the agriculture and food sector**			
Decrease the production and consumption of foods from animal sources, reduce the incidence of obesity, type 2 diabetes and cardiovascular diseases	940 (81.1)	51 (4.4)	168 (14.5)
Encourage innovation in low carbon technology and facilitate social development	958 (82.7)	56 (4.8)	145 (12.5)
Decrease the emission of air pollutants, and improve air quality as well as public health	1101 (95.0)	14 (1.2)	44 (3.8)
Increase physical activity, reduce cardiovascular diseases and obesity	929 (80.1)	88 (7.6)	142 (12.3)
**Health co-benefits of carbon emission reductions in the household sector**			
A low carbon lifestyle can conserve energy and benefit the society	1088 (93.8)	25 (2.2)	46 (4.0)
Using low carbon household appliances can promote the development of clean technology	1060 (91.5)	26 (2.2)	73 (6.3)
Low carbon lifestyle can improve people’s mental outlooks	808 (69.7)	151 (13.0)	200 (17.3)
Decrease indoor air pollutants emission, improve air quality, and promote the health of family	1083 (93.4)	23 (2.0)	53 (4.6)

**Table 5:** Multivariable logistic regression analysis for the significant predictors of respondents’ perceptions on the health co-benefits in relation to GHG mitigation.

Variables	Reference Group	OR	SE	95% CI	*p*
Age	NA (continuous)	0.98	0.01	0.97–0.99	0.020
Gender	Male	0.81	0.13	0.58–1.11	0.192
Education level	Primary school or below	1.05	0.11	0.77–1.21	0.757
Family monthly income per person (Chinese Yuan)	<1000 Chinese Yuan	1.15	0.12	0.95–1.40	0.153
Attitudes toward the current urban air pollution	Strongly disagree	1.33	0.18	1.02–1.75	0.036
Attitudes toward governmental policy attempts and progress	Strongly disagree	1.23	0.13	1.01–1.51	0.043
Have respiratory diseases	Not to have	1.50	0.31	1.01–2.25	0.047
Choose low carbon lifestyle in daily life or work	Not to choose	2.60	0.82	1.40–4.82	0.003
Constant	NA	0.02	0.01	0.00–0.09	<0.001

OR: odds ratio; SE: standard error; CI: confidence interval; ***p***: *p*-value; NA: not applicable (for reference).

## Supplementary Materials

The following are available online at www.mdpi.com/1660-4601/14/3/298/s1.
