# Supplementary material for: Perceptions of Health Co-Benefits in Relation to Greenhouse Gas Emission Reductions: A Survey among Urban Residents in Three Chinese Cities
Source: Int J Environ Res Public Health. 2017 Mar 13;14(3):298. doi: 10.3390/ijerph14030298 (PMC5369134; doi:10.3390/ijerph14030298)
Supplement: Supplementary file 1 [file ijerph-14-00298-s001.pdf]

Supplementary Materials

Perceptions of health co-benefits in relation to greenhouse gas emission reductions: A survey among urban residents in three cities, China

Jinghong Gao [1], Guozhang Xu [2], Wenjun Ma [3], Yong Zhang [4], Alistair Woodward [5], Sotiris Vardoulakis [6], Sari Kovats [7], Paul Wilkinson [8], Tianfeng He [2], Hualiang Lin [3], Tao Liu [3], Shaohua Gu [2], Jun Wang [1], Jing Li [1, 9], Jun Yang [1], Xiaobo Liu [1], Jing Li [10], Haixia Wu [1], Qiyong Liu [1, *]

1. State Key Laboratory of Infectious Disease Prevention and Control, Collaborative Innovation Center for Diagnosis and Treatment of Infectious Diseases, National Institute for Communicable Disease Control and Prevention, Chinese Center for Disease Control and Prevention, Beijing 102206, China; gaojinghong2007@126.com (J.G.); wangjun@icdc.cn (J.W.); lijingsddx@126.com (J.L.); smart_yjun@163.com (J.Y.); liuxiaobo@icdc.cn (X.L.); wuhaixia@icdc.cn (H.W.)
2. Ningbo Municipal Center for Disease Control and Prevention, Ningbo 315010, China; xugz@nbcdc.org.cn (G.X.); hetf@nbcdc.org.cn (T.H.); gushaohua1989@126.com (S.G.)
3. Guangdong Provincial Institute of Public Health, Guangdong Provincial Center for Disease Control and Prevention, Guangzhou 511430, China; mawj@gdiph.org.cn (W.M.); linhualiang2002@163.com (H.L.); gztt_2002@163.com (T.L.)
4. Beijing Center for Disease Control and Prevention, Beijing 100013, China; ZY968919@sina.com
5. School of Population Health, University of Auckland, Private Bag 92019, Auckland, New Zealand; a.woodward@auckland.ac.nz
6. Environmental Change Department, Centre for Radiation, Chemical and Environmental Hazards, Public Health England, Chilton OX11 0RQ, UK; sotiris.vardoulakis@phe.gov.uk
7. NIHR Health Protection Research Unit in Environmental Change and Health, London School of Hygiene and Tropical Medicine (LSHTM), 15-17 Tavistock Place, London WC1H 9SH, UK; sari.kovats@lshtm.ac.uk
8. Public and Environmental Health Research Unit, London School of Hygiene and Tropical Medicine, 15-17 Tavistock Place, London WC1H 9SH, UK; paul.wilkinson@lshtm.ac.uk
9. Department of Epidemiology, School of Public Health, Shandong University, Jinan 250012, China
10. Changping District Centre for Disease Control and Prevention, Beijing 102200, China; lijing986169323@sina.cn

* Correspondence: liuqiyong@icdc.cn (Q.L.); Tel.: +86-10-58900740; Fax: +86-10-58900739

Availability of materials and survey instrument

The full version of the survey instrument used during the current study are not publicly available due to the requirements of the project supported the present study and the fact that the instrument are still important materials of our forthcoming companion papers on the topic of climate change, air pollution, and low carbon transition, but they are available from the corresponding author on reasonable request. The following supplementary materials only provide a subset of the questionnaire involving questions on the health co-benefits of GHG emission reductions, which are the focus of the present study.

Correspondence and requests for materials should be addressed to Q. L. (liuqiyong@icdc.cn)

Questionnaire for Field Survey

Part C. Health co-benefits of carbon emission reductions

1. Do you personally follow low carbon lifestyle in your daily life or work routine?

（1）Yes, please list some examples:________________________

（2）No, I do not choose low carbon lifestyle. *(If you choose this option, please skip question 2)*

2. Do you think your low carbon lifestyle have positive impact on your personal health?

（1）Yes

（2）No

3. Have you ever heard about the health co-benefits of carbon emission reductions?

（1）Yes, and I am familiar with the meanning of the concept

（2）Yes, but I do not know the specific content of the concept

（3）No, I have never heard about it

4. Reducing carbon emissions can not only mitigate climate change but also bring about health co-benefits through various channels.

（1）Strongly disagree

（2）Disagree

（3）Neutral

（4）Agree

（5）Strongly agree

5. Through which pathways could carbon emission reductions create co-benefits for public health? **(Multiple answers)**

（1）Reduce indoor and outdoor air pollution and improve air quality

（2）Improve mental outlook

（3）Increase the amount of physical activities

（4）Reduce the intake of unhealthy or junk food (e.g. food with high fat content)

（5）Improve the living, producing (built) and ecological environment

（6）Other:________________________

6. In energy production and use, through what ways could carbon emission reductions bring about health co-benefits?

(1) Increase physical activity, reduce obesity and cardiovascular diseases	Yes	No	Uncertain
(2) Mitigate climate change, decrease the burden of climate-sensitive diseases	Yes	No	Uncertain
(3) Encourage scientific innovation and facilitate social development	Yes	No	Uncertain
(4) Decrease air pollutants, improve air quality, and reduce diseases caused by air pollution	Yes	No	Uncertain
(5) Other:	Yes	No	Uncertain

7. In transport system, through what ways could carbon emission reductions bring about health co-benefits?

(1) Improve the quality of vehicles and decrease road traffic injuries	Yes	No	Uncertain
(2) Promote the development and use of low carbon and environmental friendly vehicles	Yes	No	Uncertain
(3) Improve physical activities, decrease cardiovascular diseases, obesity and diabetes through promoting active travel (walking, cycling and public transport)	Yes	No	Uncertain
(4) Decrease vehicle use and air pollutants emission, improve air quality and public health	Yes	No	Uncertain
(5) Other:	Yes	No	Uncertain

8. In agriculture and food sector, what health co-benefits could carbon emission reductions bring about?

(1) Decrease the production and consumption of foods from animal sources, reduce the incidence of obesity, type 2 diabetes and cardiovascular diseases	Yes	No	Uncertain
(2) Encourage innovation in low carbon technology and facilitate social development	Yes	No	Uncertain
(3) Decrease the emission of air pollutants, and improve air quality as well as public health	Yes	No	Uncertain
(4) Increase physical activity, reduce cardiovascular diseases and obesity	Yes	No	Uncertain
(5) Other:	Yes	No	Uncertain

9. In household sector, what health co-benefits could carbon emission reductions bring about?

(1) A low carbon lifestyle can conserve energy and benefit the society	Yes	No	Uncertain
(2) Using low carbon household appliances can promote the development of clean technology	Yes	No	Uncertain
(3) Low carbon lifestyle can improve people's mental outlooks	Yes	No	Uncertain
(4) Decrease indoor air pollutants emission, improve air quality, and promote the health of family	Yes	No	Uncertain
(5) Other:	Yes	No	Uncertain

Part E. Socio-demographic Information

10. Your gender is:
（1）Male （2）Female

11. What is your ethnicity?
（1）Han （2）Hui （3）Other:____________

12.Your age or date of birth:____________

13.What is your education level？
（1）Primary school or below
（2）Junior middle school
（3）Senior middle or vocational school
（4）Bachelor degree
（5）Master degree or above

14. Where is your residence registered (Hukou)?
（1）Urban （2）Rural

15. How long have you lived (worked) in Beijing/Ningbo/Guangzhou?
Years:____________

16. What is your marital status?
（1）Unmarried （2）Married （3）Widowed （4）Divorced

17. Your occupation is:
（1）Worker （2）Farmer/Fisher/Agriculture related
（3）Police/Army （4）Teaching staff
（5）Government staff （6）Student
（7）Commerce or service trade （8）Medical personnel
（9）Technician （10）Company employee
（11）Self-Employed （12）Retired
（13）Unemployment （14）Other:____________

18.What is your current family average monthly income (including wage/salary, subsidy, and welfare) (Chinese yuan)?
（1）< ¥1000 （2）¥1000 - ¥2000 （3）¥2000 - ¥3000
（4）¥3000 - ¥5000 （5）¥5000 - ¥10000 （6）>¥10000

19. How about your health status?
（1）Very poor （2）Relatively poor （3）Average
（4）Relatively good （5）Very good

20. Have you ever been diagnosed with the following health conditions? **(Multiple answers)**

Cardiovascular Diseases：

（1）Hypertension （2）Coronary Heart Disease

（3）Angina （4）Stroke

（5）Other cardiovascular/cerebral diseases：_________

Respiratory Diseases：

（6）Emphysema （7）Chronic bronchitis

（8）Asthma （9）Other respiratory illnesses：_________

Other Diseases：

（10）Chronic Nephritis （11）Diabetes

（12）Cancer （13）Disability

（14）Other diseases：_____________

（15）I have never been diagnosed with any of the above diseases

If you are willing, you could provide your family name and your contact information (QQ, Wechat, email address or phone number), so that we could send the result of the survey to you. If you are inconvenience and not willing, that is completely fine as well.

Family Name：_______________

Contact information：________________

End of the Questionnaire

Thank you for your support and cooperation

Investigator：____________ **Inspector：**____________ **Date：**____________
